# Supplementary material for: Close-packed polymer crystals from two-monomer-connected precursors
Source: Nat Commun. 2016 Sep 19;7:12803. doi: 10.1038/ncomms12803 (PMC5031798; doi:10.1038/ncomms12803)
Supplement: Supplementary Information — Supplementary Figures 1-10 and Supplementary Reference [file ncomms12803-s1.pdf]

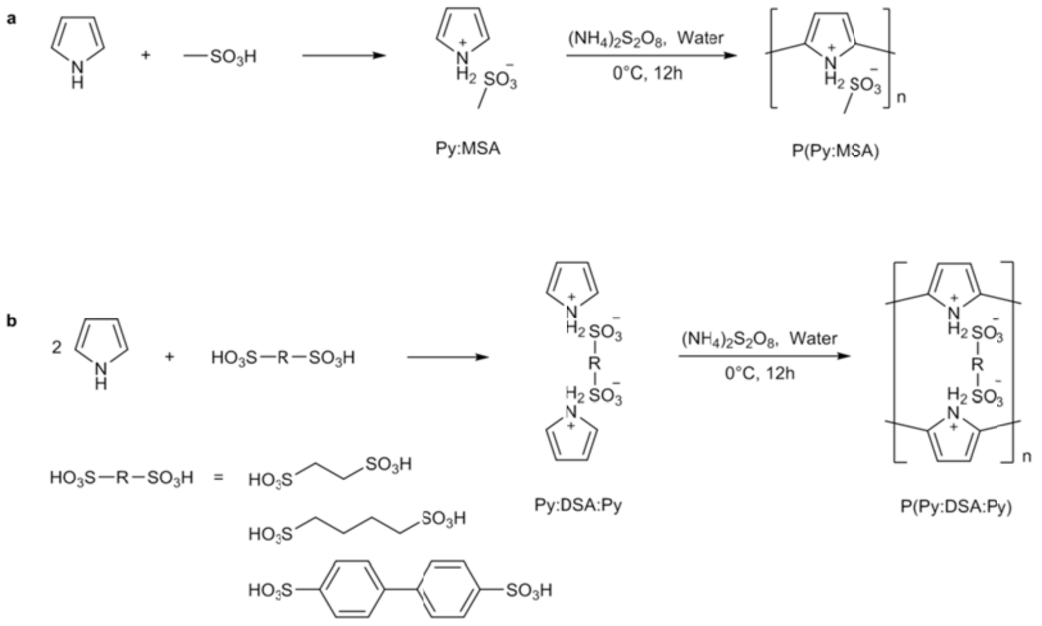

Supplementary Figure 1 | Synthesis of P(Py:MSA) and P(Py:DSA:Py)s. Synthetic scheme of P(Py:MSA) (**a**) and P(Py:DSA:Py)s (**b**).

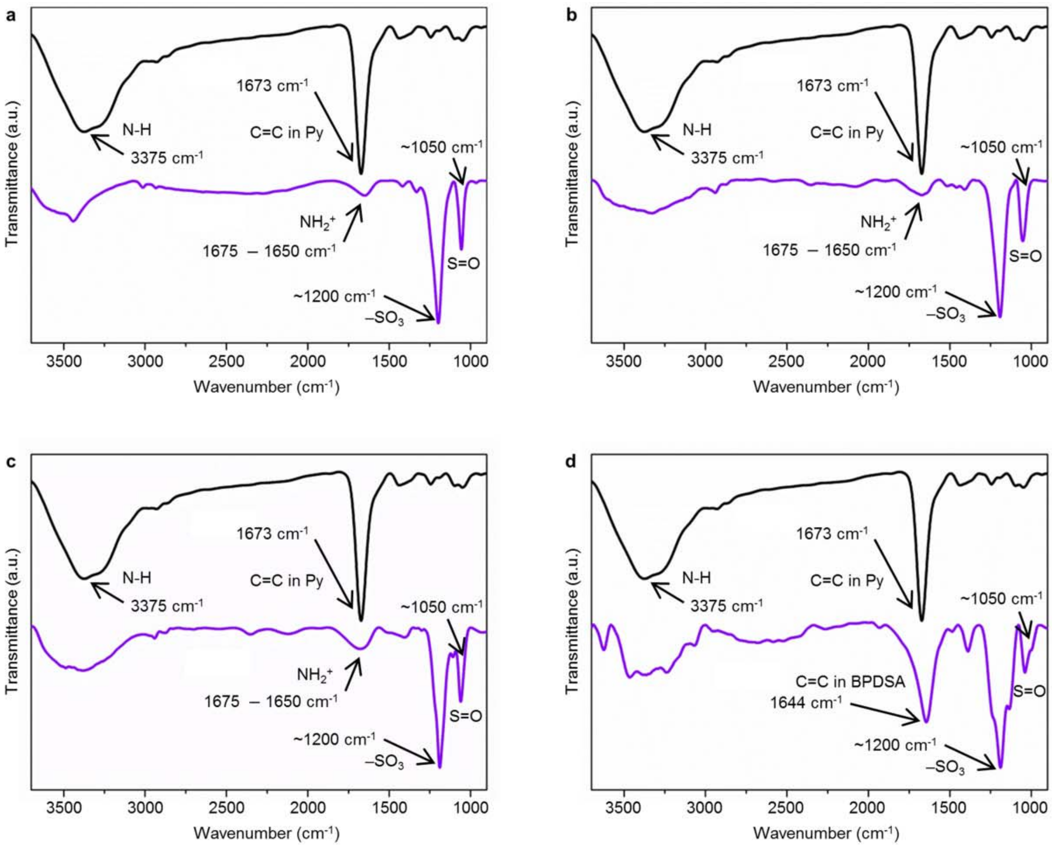

Supplementary Figure 2 | FT-IR spectra of Py:MSA and TMCPs. The black line in each graph is the FT-IR spectrum of Py. (**a-d**) FT-IR spectra (violet graphs) of Py:MSA (**a**), Py:EDSA:Py (**b**), Py:BDSA:Py (**c**) and Py:BPDSA:Py (**d**). In the FT-IR spectrum of Py, a strong and broad peak at 3375 cm^{-1}, which is associated with the N–H stretching vibration[1], is observed. The peak at 1673 cm^{-1} corresponds to C=C stretching in Py ring[1]. However, in the case of the Py salts, the N–H band is decreased and shifted. In addition, no C=C stretching in the Py ring is observed in the spectra. By contrast, the vibration band in the 1675–1650 cm^{-1} region, which is believed to be associated with the NH^{2+} deformation band[1], is observed in the spectra of all Py salt samples. The strong peak at 1644 cm^{-1} in the spectrum of Py:BPDSA:Py (**d**) corresponds to C=C stretching[1] in the biphenyl ring in the connector. Below 1500 cm^{-1}, the peaks observed near 1200 cm^{-1} and 1050 cm^{-1} are attributed to the stretching vibrations of the $–SO_3$ group and S=O, respectively[1].

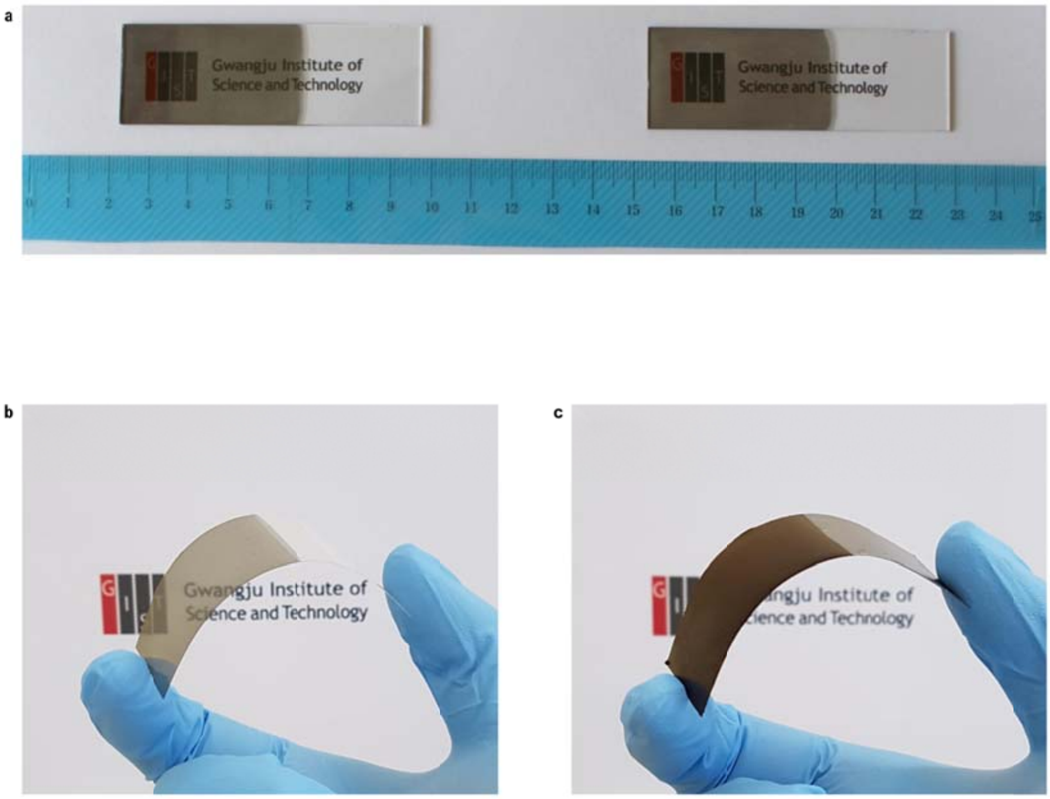

Supplementary Figure 3 | Photographs of *in situ* polymerized P(Py:EDSA:Py) films on various substrates. The size of the P(Py:EDSA:Py) films was 4.5 × 2.5 cm (film area: 11.25 cm^2). The thickness of the P(Py:EDSA:Py) films, which were coated on both sides of the substrates, was ~150 nm. (**a**) *In situ* polymerized P(Py:EDSA:Py) films on a glass slide and an ITO-coated glass slide. (**b**) *In situ* polymerized P(Py:EDSA:Py) film on a flexible PET film. (**c**) *In situ* polymerized P(Py:EDSA:Py) film on a flexible graphite foil.

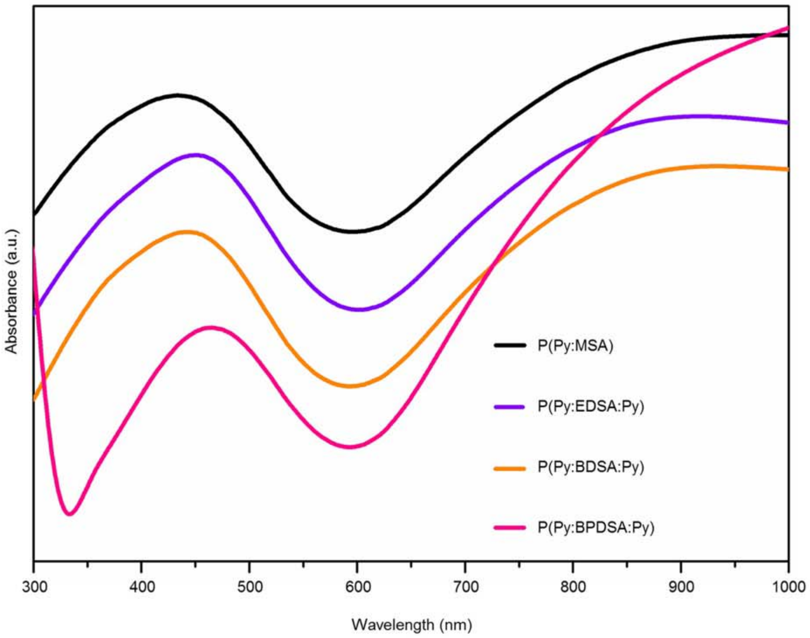

Supplementary Figure 4 | UV-vis absorption spectra of P(Py:MSA) and P(Py:DSA:Py) films. UV-vis absorption spectra of P(Py:MSA) and P(Py:DSA:Py) films coated on optically clear glass substrates via *in situ* polymerization, where the spectral features depend on the ordered structures of the polymer chains. The maximum absorption peaks of the P(Py:DSA:Py) films are redshifted compared with that of P(Py:MSA).

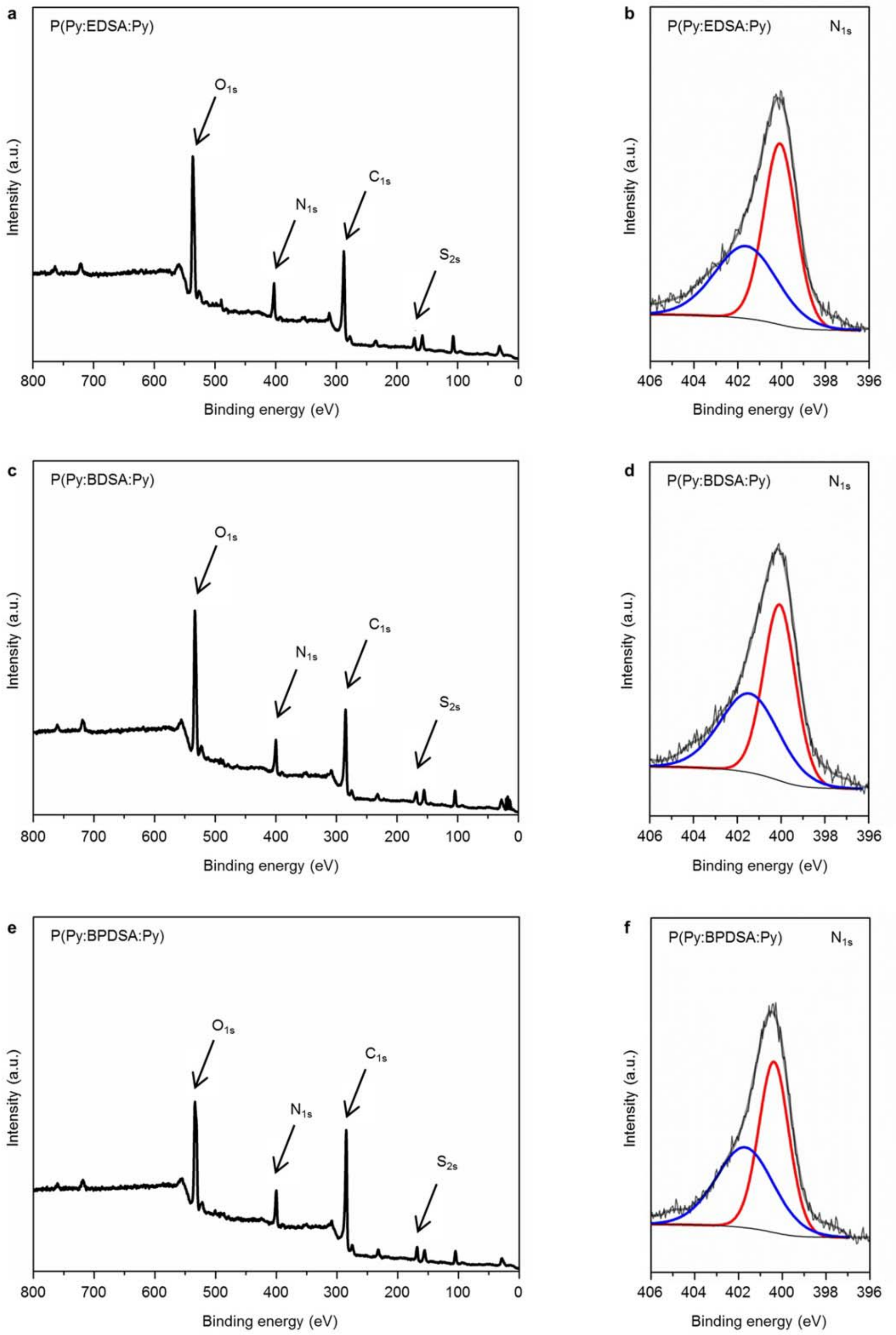

Supplementary Figure 5 | XPS spectra of P(Py:DSA:Py) films. (**a,c,e**) The full spectra of P(Py:DSA:Py) (**a**), P(Py:BDSA:Py) (**c**), and P(Py:BPDSA:Py) (**e**). (**b,d,f**) The N_{1s} signals of P(Py:EDSA:Py) (**b**), P(Py:BDSA:Py) (**d**), and P(Py:BPDSA:Py) (**f**). The peaks indicate the presence of charged Py in the polaron (–$NH^{\bullet+}$–, red) and bipolaron (=NH^{+}–, blue) states.

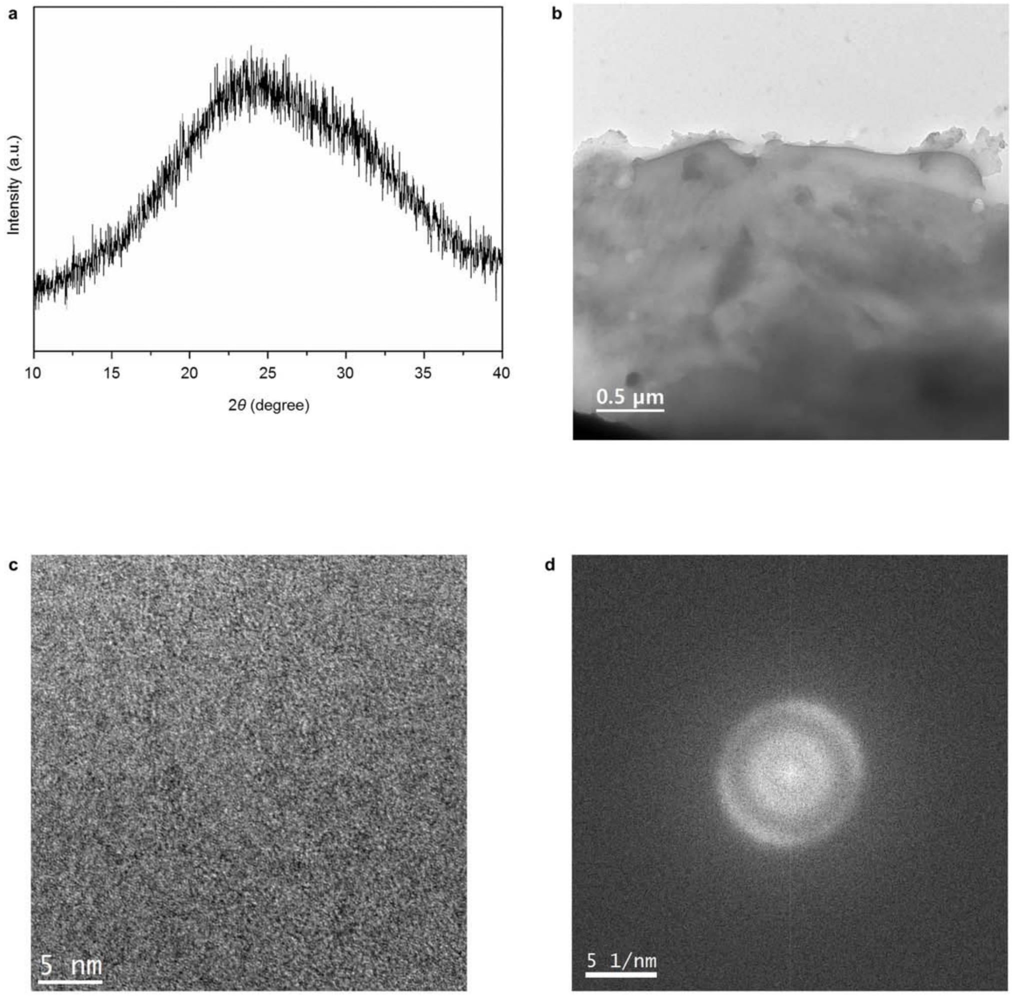

Supplementary Figure 6 | XRD and TEM micrographs of P(Py:MSA). (**a**) XRD spectrum. (**b**) Low-magnification bright-field (BF) TEM image. (**c**) HRTEM image. (**d**) FFT image.

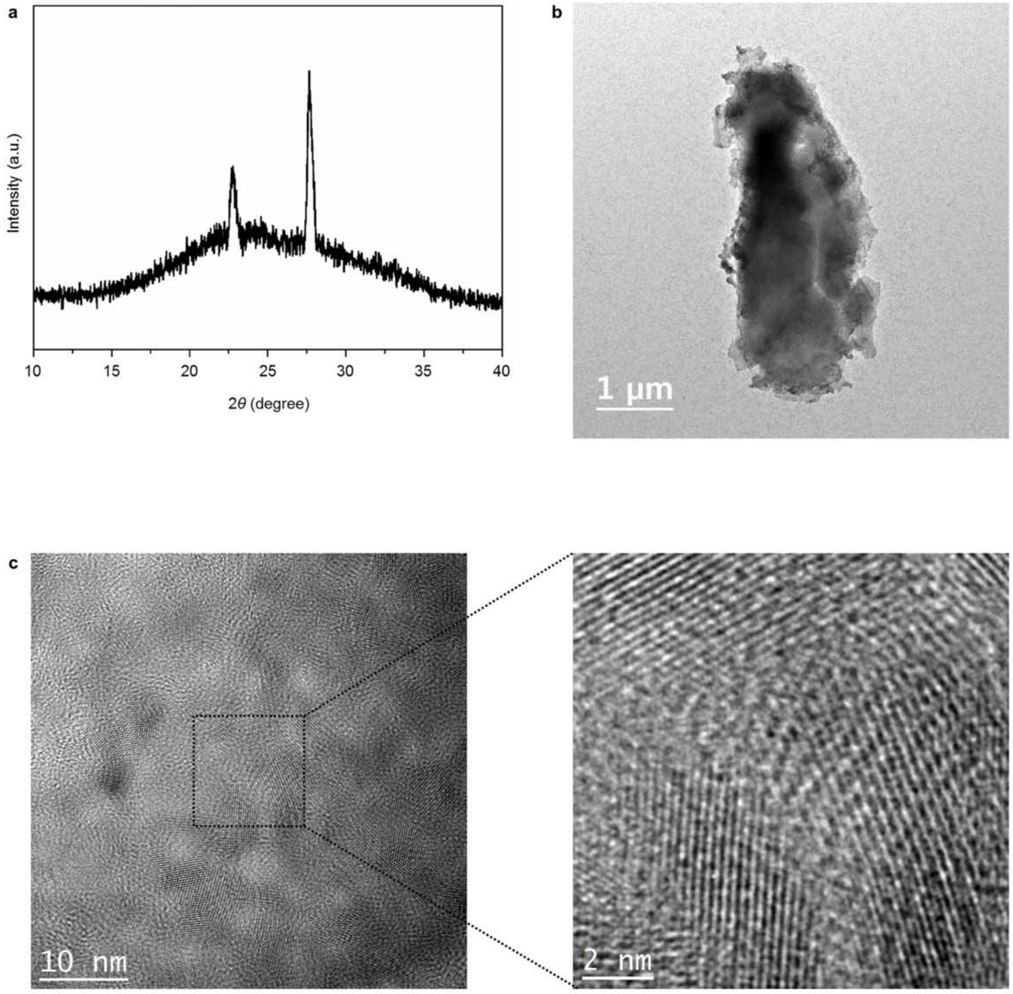

Supplementary Figure 7 | XRD and TEM micrographs of P(Py:BDSA:Py). (**a**) XRD spectrum. (**b**) Low-magnification BF TEM image. (**c**) HRTEM images.

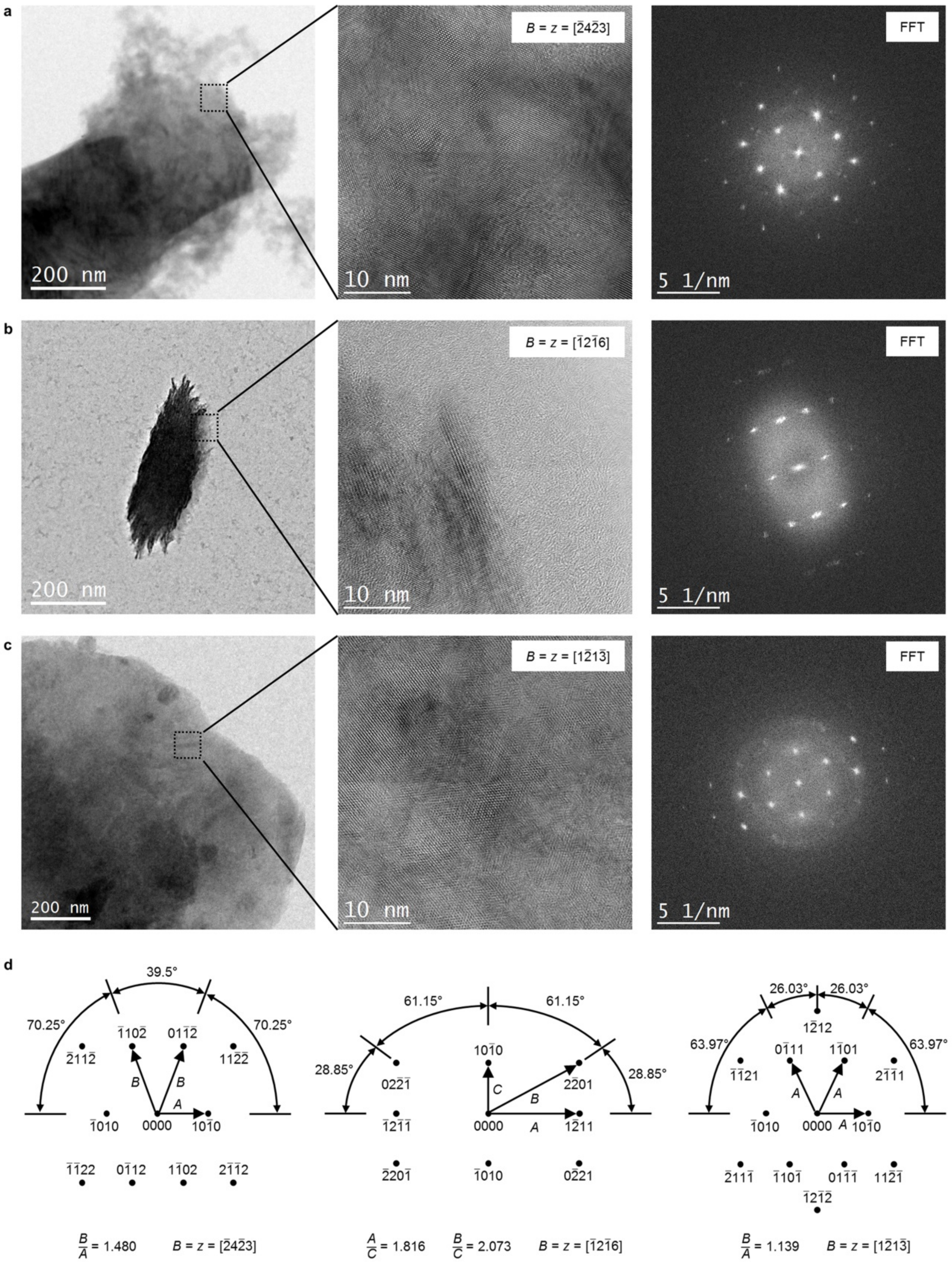

Supplementary Figure 8 | Characterization of the crystal structure of P(Py:EDSA:Py). (**a-c**) HRTEM and FFT images of P(Py:EDSA:Py) measured along the $[\bar{2}4\bar{2}3]$ direction (**a**), $[\bar{1}2\bar{1}6]$ direction (**b**), and $[1\bar{2}1\bar{3}]$ direction (**c**). (**d**) Theoretical transmission electron diffraction patterns confirming the HCP crystal structure.

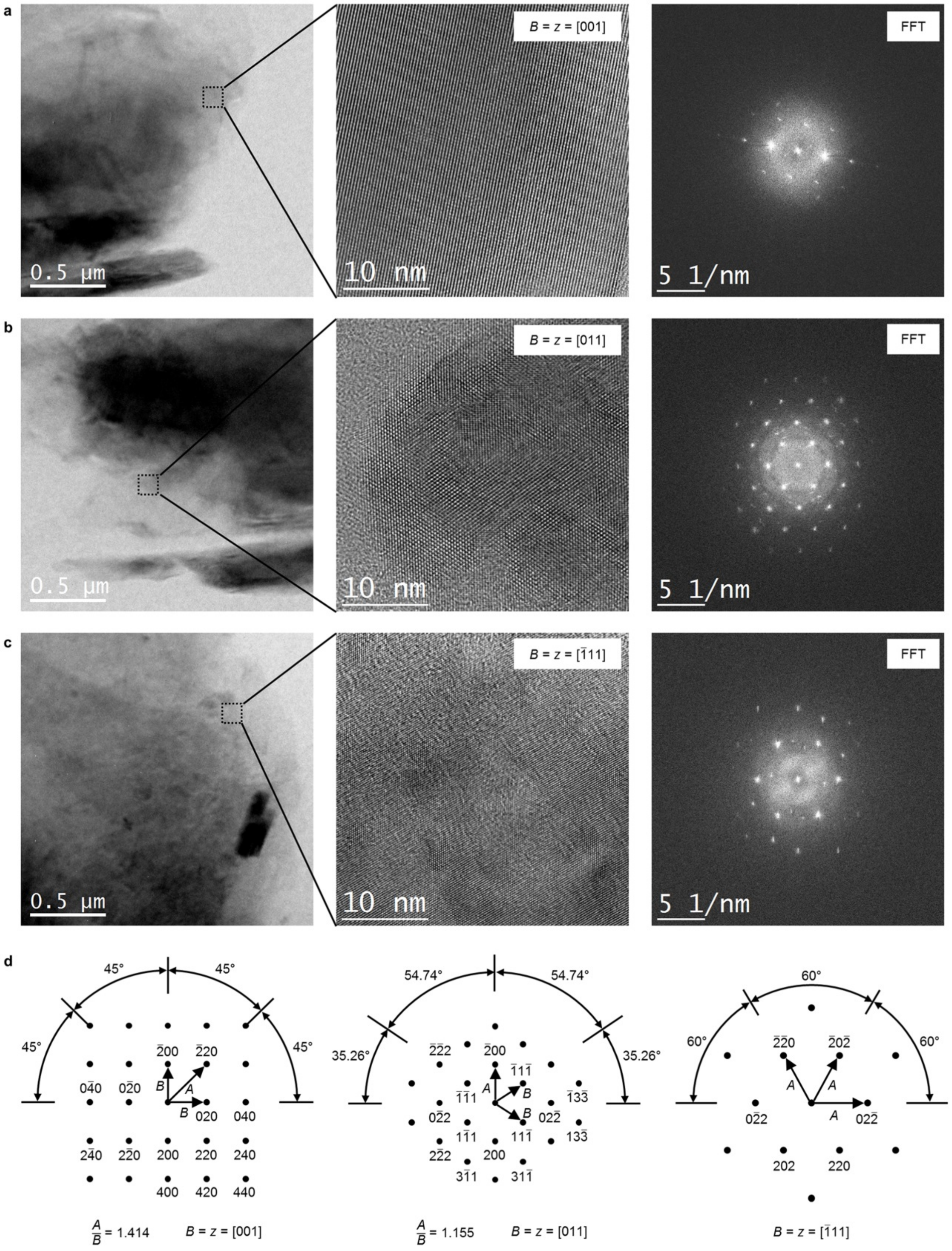

Supplementary Figure 9 | Characterization of the crystal structure of P(Py:BPDSA:Py). (**a-c**) HRTEM and FFT images of P(Py:BPDSA:Py) measured along the [001] direction (**a**), [011] direction (**b**), and [$\bar{1}$11] direction (**c**). (**d**) Theoretical transmission electron diffraction patterns confirming the FCC crystal structure.

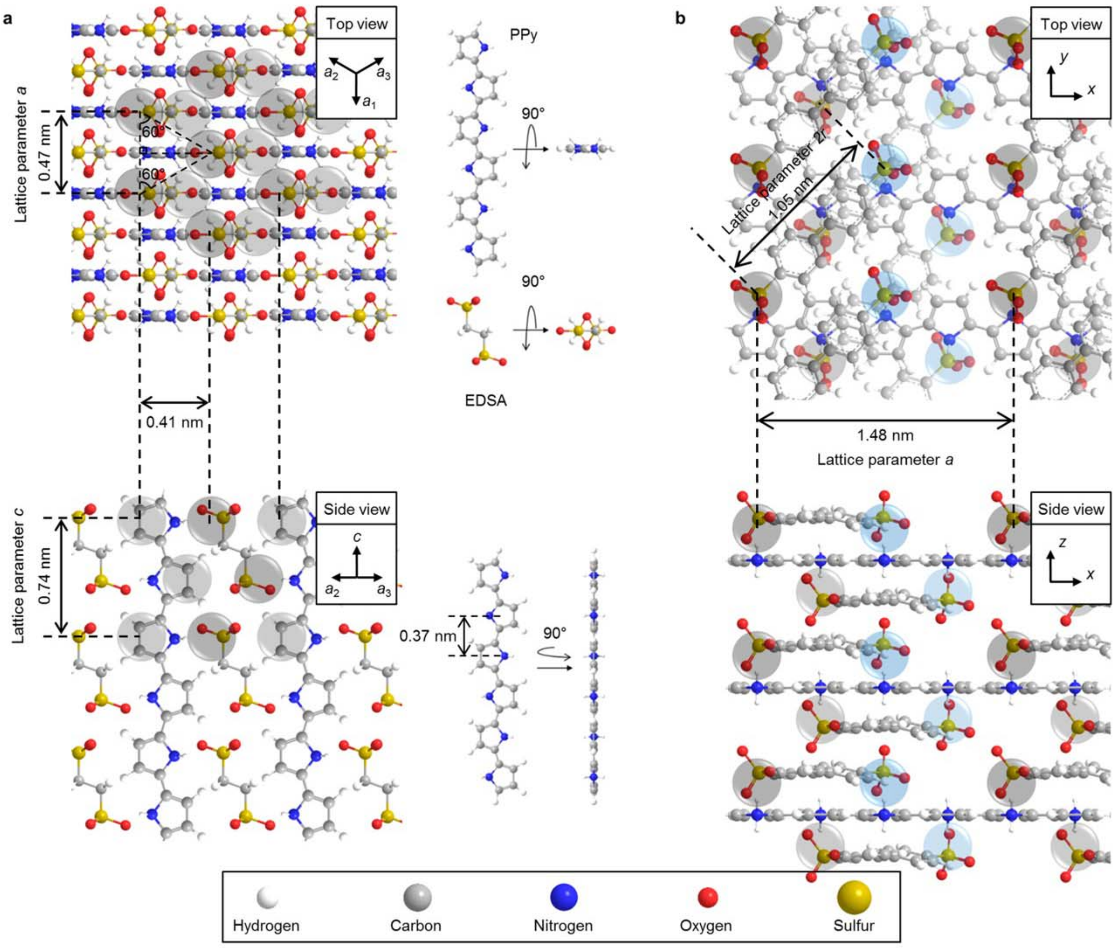

Supplementary Figure 10 | Schematic representations of proposed chemical arrangements of P(Py:EDSA:Py) and P(Py:BPDSA:Py). The schematic representations were drawn on the basis of the experimental results of TEM, XRD and XPS. (**a**) Top and side views of the crystal structure of P(Py:EDSA:Py). Ammonium-sulfonate ionic clusters (grey circles) are hexagonally packed in the (0001) plane. (**b**) Top and side views of the crystal structure of P(Py:BPDSA:Py). Ammonium-sulfonate ionic clusters (grey and blue circles) are cubically ordered with a layered rock-salt structure.

Supplementary Reference

1. Patnaik, P. *Dean's analytical chemistry handbook* 2nd edn (McGraw-Hill, 2004).
